# Comparison of spring characteristics of titanium-molybdenum alloy and stainless steel

**DOI:** 10.4317/jced.532741

**Published:** 2018-06-01

**Authors:** Ahmad Sheibaninia, Anahita Salehi, Armen Asatourian

**Affiliations:** 1 DDS, MS, Associate Professor, Fellowship of Orthosurgery, Department of Orthodontic, Islamic Azad University, Dental School, Tehran Branch, Tehran, Iran; 2 DDS, MS, Private Practice, UAE; 3 DDS, Clinical instructor, Angiogenesis and Regenerative Group, Dr. H. Afsar Lajevardi Research Cluster, Shiraz, Iran

In this article by Sheibaninia and colleagues ( *J Clin Exp Dent* 2017;9: 84-90 1 Jan), there is a conflict among the authors. The editor decided to withdraw the article.
